# DeepFruit: A dataset of fruit images for fruit classification and calories calculation

**DOI:** 10.1016/j.dib.2023.109524

**Published:** 2023-08-28

**Authors:** Ghazanfar Latif, Nazeeruddin Mohammad, Jaafar Alghazo

**Affiliations:** a Department of Computer Science, Prince Mohammad bin Fahd University, Al Khobar, Saudi Arabia; b Department of Computer Sciences and Mathematics, Université du Québec à Chicoutimi, 555 boulevard de l'Université, Québec, Canada; c Cybersecurity Center, Prince Mohammad bin Fahd University, Al Khobar, Saudi Arabia; d Artificial Intelligence Research Initiative, College of Engineering and Mines University of North Dakota, North Dakota, United States

**Keywords:** Multiple fruits dataset, Calorie estimation, Fruits images, Fruits classification, Agriculture produce, Artificial intelligence, Pattern recognition, Machine learning

## Abstract

A dataset of fully labeled images of 20 different kinds of fruits is developed for research purposes in the area of detection, recognition, and classification of fruits. Applications can range from fruit recognition to calorie estimation, and other innovative applications. Using this dataset, researchers are given the opportunity to research and develop automatic systems for the detection and recognition of fruit images using deep learning algorithms, computer vision, and machine learning algorithms. The main contribution is a very large dataset of fully labeled images that are publicly accessible and available for all researchers free of charge. The dataset is called “DeepFruit”, which consists of 21,122 fruit images for 8 different fruit set combinations. Each image contains a different combination of four or five fruits. The fruit images were captured on different plate sizes, shapes, and colors with varying angles, brightness levels, and distances. The dataset images were captured with various angles and distances but could be cleared by utilizing the preprocessing techniques that allow for noise removal, centering of the image, and others. Preprocessing was done on the dataset such as image rotation & cropping, scale normalization, and others to make the images uniform. The dataset is randomly partitioned into an 80% training set (16,899 images) and a 20% testing set (4,223 images). The dataset along with the labels is publicly accessible at: https://data.mendeley.com/datasets/5prc54r4rt.

Specifications Table

**Table:** 

Subject	Computer Vision and Pattern Recognition
Specific subject area	Internet of Things (IoT), Fruit type recognition, Computer Science Applications, Computer Vision and Pattern Recognition, Machine Learning, Deep Learning, and Artificial Intelligence.
Type of data	Images (64 × 64 pixels JPG format) Figures CSV files
How data were acquired	Images are captured using smartphone Samsung Galaxy S10
Data format	Analyzed
Description of data collection	20 different fruit types were collected, and various plates were used. Fruits were organized on plates according to 8 different predetermined combination sets while varying the arrangements during the process. Pictures were taken of the arranged plates from a distance of around 25–50 cm with enough brightness. The result is a dataset consisting of 21,122 JPG images for 20 different fruit types (classes) and 8 different combination sets of fruits. Each image has a combination of four or five different fruits. The JPG images are fully labeled and shown in Table 1.
Data source location	Institution: Prince Mohammad bin Fahd University City/Town/Region: Al Khobar, Eastern Province Country: Saudi Arabia. Latitude and longitude: 26.307580, 50.198940
Data accessibility	**Repository name:** Mendeley Data **Direct URL to data:**https://data.mendeley.com/datasets/5prc54r4rt **DOI:**10.17632/5prc54r4rt.1′

## 1 Value of the data

• This dataset is useful for fruit recognition and calorie estimation from the images, which can be helpful for diet control [1], [2], [3]. This dataset contains images of different combinations of fruits, which makes it possible to develop multi-type fruit identification models. These models can be used in health monitoring applications to observe fruit intake and calorie estimation.
• The data can be used by machine learning researchers/companies to develop models for recognizing different fruits [4], [5].
• The current research trends in deep learning and machine learning target mainly the development of applications for everyday use such as face recognition, fingerprint recognition, or application in the fields of healthcare, engineering, and many others. Image recognition applications usually go through different phases starting from preprocessing to recognition for autonomous tasks that are usually done by persons. Preprocessing may consist of processes such as data cleaning, dimensionality reduction, resizing, and labeling. Recognition is the process of categorizing the objects of interest. This is a general process that is used in machine learning. The Fruits images dataset can be used or reused as a unique resource for researchers working on the development of applications for fruit recognition, daily diet intake plans, education, and learning about nutrition facts in different fruits and other related applications using machine learning approaches.
• The DeepFruit dataset with different combinations is a comprehensive repository of fully labeled 20 different types of fruit images to develop automated applications related to fruit recognition and diet plans.
• The Fruits images dataset serves as a base for researchers to enhance and develop this dataset further by producing more images with more variations. Researchers can further add more classes to the dataset, increase the number of images per class, increase the complexity of the images, etc. The researchers will develop algorithms and applications that can be used by consumers for dietary purposes or any other purpose.
• Using existing nutritional databases, it is easy to calculate the calories when the number and types of fruit are identified as every fruit used to have specific calories [6]. The following are general steps to calculate the calories of a fruit plate: 1. Determine the types and number of fruits in the fruit plate; 2. Use a calorie database and find the calorie content of each type of fruit in the fruit plate; 3. Multiply the calorie content of each fruit portion size of the fruit in the plate; 4. Add up the calorie values of all the fruits in the fruit plate to get the total calorie count of the plate.
  ○ For example, if a fruit plate contains 1 pear (49 calories), 2 medium apple (95 calories), and 3 medium banana (105 calories), the total calorie count of the fruit plate would be: 49 + 2 × 95 + 3 × 105 = 554 calories.

## 2 Objective

The existing Fruits dataset (Fruits 360 [7]) contains a smaller number of images and does not have variations and did not contain multiple fruit sets in the single images. In recent literature [1], [2], [3], [4], [5], fruit recognition is mainly done using single fruit in an image, and does not contain multiple types of fruits in a single image. Therefore, there is a need to develop a comprehensive dataset containing a variety of fruits with different fruit set combinations captured on different plates’ sizes, shapes, and colors with varying angles, brightness levels, and distances. Further, the fruit datasets used in the recent studies are not publicly available for future research.

## 3 Data description

**Fig. 1:**
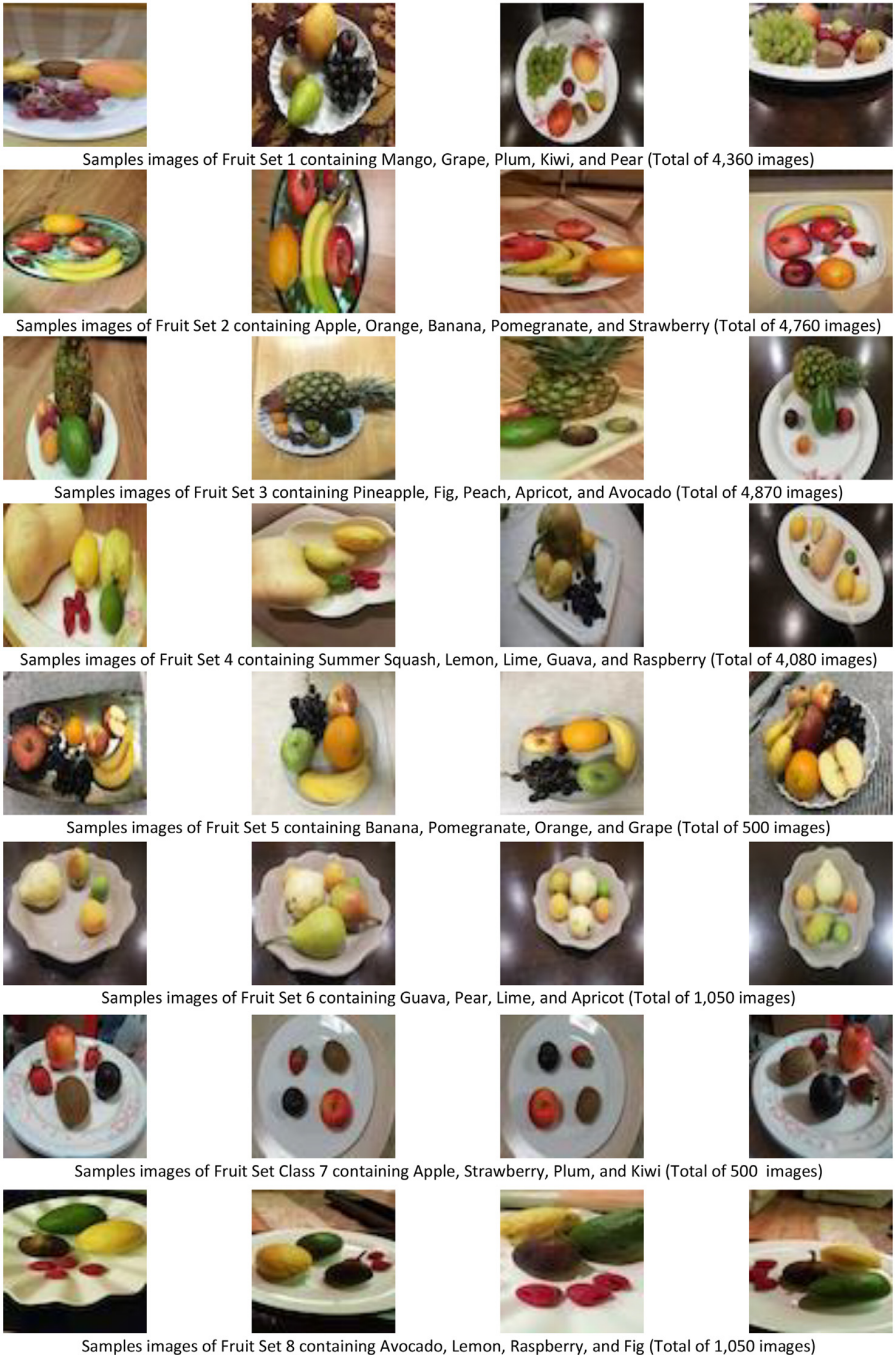
Sample fruit images from the dataset with different combination sets (total of 21,122 images).

The Fruit images dataset (DeepFruit) is a newly developed labeled dataset which is developed at Prince Mohammad Bin Fahd University, Al Khobar, Kingdom of Saudi Arabia. DeepFruit dataset for the fruit images is developed using the most commonly available 20 fruits in Saudi supermarkets. The dataset is publicly available in [7] and consists of 21,122 images. Table 1 shows the different classes with their corresponding fruit set class, names, and the total number of images. The number of classes 20 corresponding to the 20 most common fruit types were named with the fruit class each represented as shown in Table 1 (column 1). The dataset consists of different sets of fruits in each image and examples of each set are shown in Fig. 1, which are also summarized in Table 1 (column 2). A fruit type may appear in more than one set. For example, as can be seen from Fig. 1, “Grape” is part of Set 1 and Set 5, thus, Grape has Fruit Set ID 1, 5 in Table (column 2). The total number of captured images for each fruit class is shown in Table 1 (column 3).

**Table 1:** Summary of the newly constructed Fruits Images dataset (DeepFruit).

Fruit Class	Fruit Set ID	Total Images
Mango	1	4360
Grape	1, 5	4860
Plum	1, 7	4860
Kiwi	1, 7	4860
Pear	1, 6	5410
Apple	2, 7	5260
Orange	2, 5	5260
Banana	2, 5	5260
Pomegranate	2, 5	5260
Strawberry	2, 7	5260
Pineapple	3	4870
Fig	3, 8	5920
Peach	3	4870
Apricot	3, 6	5920
Avocado	3, 8	5920
Summer Squash	4	4080
Lemon	4, 8	5130
Lime	4, 6	5130
Guava	4, 6	5130
Raspberry	4, 8	5130

The newly developed dataset consists of 21,122 fruit images randomly partitioned into an 80% training set (16,899 images) and a 20% testing set (4223 images) as can be found in [8]. The uploaded dataset contains the “Fruits_Dataset_Test” directory has the fruit images for testing and their labels are provided in “Labels_Test.csv”. The first column of each row of this file contains the image file Name and the next 20 columns are for 20 fruit types where cell value 1 represents fruit is in the image and 0 means particular fruit is not in the image. For example, the first row has the image File name “IMG_20,190,107_171,401_1.jpg ” and contains 5 types of Fruits (Mango, Grape, Plum, Kiwi, and Pear) as shown in Columns 2 to 6 with cell values 1 while columns 7 to 21 has values 0 means those fruits are not present in the image. Similarly, the “Fruits_Dataset_Train” directory has the fruit images for training and their labels are provided in “Labels_Train.csv”. The “Sample_Images” directory contains sample fruit images for a quick review before downloading the complete dataset.

The dataset is comprehensive and was proven to be sufficient for training and classification based on the initial accuracy results of 94.72% using the Convolutional Neural Network model (GoogleNet) [9] and detailed experimental results will be published in a separate journal/conference paper. The current version of the published dataset can be used as is, while future versions may include more image variations, fruit types and numbers. The fruits images dataset is not without limitations and the insufficient light, as well as the non-inclusion of all the Fruit classes that exist as we only limited our dataset to 20 different types of fruits from available fruits, are limitations that will be addressed in future versions of the dataset.

## 4 Experimental design, materials, and methods

The DeepFruit is a fully labeled Fruits images dataset captured with different combination sets. The development of the dataset was done at Prince Mohammad Bin Fahd University. Students volunteered in capturing and labeling the images (under the supervision of the authors), which are taken using a smart digital phone camera (Samsung Galaxy S10) with the default setting; resolution 3840 × 2160 and JPG image format. The authors also double-checked each image after the students to ensure that they are within the scope and specifications specified for this research. The images were taken at different times of the day with different light conditions (bright light, low light, backlighting, with flash, and without flash.), positions, and distances (between 1 and 2 feet from the fruit plate). The total number of images per Fruit type is different, however, the dataset consists of 21,122 images in total. The newly developed dataset is stored as 3-channel RGB images with different dimensions and variations.

Following are the steps that can be followed to reproduce the dataset:

1. Collect different fruits (refer to Table 1).
2. Collect different plates with varying designs and sizes.
3. Place the fruits randomly on different plates with varying types of fruits (3 to 5 types of fruits).
4. Use a smartphone (such as Samsung Galaxy S10) to capture fruit images.
5. The images need to be captured with varying positions, distances from the camera, and light conditions (bright light, low light, backlighting, with flash, without flash, etc.).

## Ethics statements

The collected data does not involve human subjects or animals as well as the data is not collected from social media platforms.

## CRediT authorship contribution statement

**Ghazanfar Latif:** Conceptualization, Methodology, Supervision. **Nazeeruddin Mohammad:** Data curation, Writing – original draft. **Jaafar Alghazo:** Visualization, Writing – review & editing.

## Data Availability

DeepFruits: Dataset of Fruits Images with different combinations for Fruit Recognition and Calories Estimation (Original data) (Mendeley Data).
